# Magnitude and associated factors of thrombocytopenia, among pregnant women at Mizan Tepi university teaching hospital south west Ethiopia

**DOI:** 10.1186/s12884-024-06609-6

**Published:** 2024-06-07

**Authors:** Samuel Sahile Kebede, Tariku Daniel, Asamrew Alemu, Getachew Mesfin Bambo

**Affiliations:** 1 Department of Medical Laboratory sciences, College of Medicine and Health Sciences, Mizan Tepi University, Mizan, Ethiopia; 2 Department of Nursing, College of Medicine and Health Sciences, Mizan Tepi University, Mizan, Ethiopia

**Keywords:** Associated factors, Antenatal care, Gestational thrombocytopenia, Pregnancy

## Abstract

**Background:**

Thrombocytopenia in pregnancy is a common multifactorial abnormality of the hematological system, next to anemia. It leads to more increased risk of bleeding during delivery, labour, or the postpartum period. Despite being a significant public health concern, there are limited studies done concerning thrombocytopenia during pregnancy.

**Objective:**

To assess the magnitude and associated factors of thrombocytopenia among pregnant women at Mizan Tepi University Teaching Hospital from September 2023 to November 2023.

**Methods:**

An institutional-based cross-sectional study was carried out on 230 systematic randomly selected pregnant women who attended antenatal visits from September 2023 to November 2023 G.C using data collection tools. The pretested structured questionnaires were employed to obtain clinical, nutritional, and sociodemographic information. Additionally, three millilitres of venous blood were collected from each participant and analyzed using a Sysmex hematology analyzer. The data was entered into Epidata version 4.6 and analyzed using STATA version 14. Descriptive statistics were computed, and logistic regression was used to identify predictors with a significance level of less than 0.05.

**Results:**

Two hundred thirty pregnant women participated in the study. Among study participants, the magnitude of thrombocytopenia was 55(24.35%) with 32 (57.14%) mild, 19 (33.93%) moderate, and 5 (8.93%) severe thrombocytopenia. The determinant factors which shown significant association were Malaria parasite infection (AOR 9.27 at 95% CI 7.42, 10.87), one-year Inter-birth interval (AOR 1.7 at 95% CI 1.24, 2.14), History of abortion (AOR 3.94 95% CI 3.13, 4.86), History of hypertension (AOR 3.12 95% CI 1.56, 4.12), HIV infection (AOR 1.81 95% CI 1.32.2.52) and HBV infection (AOR 3.0 95% CI 2.82, 3.34).

**Conclusion:**

Thrombocytopenia is a public health problem and mild type of thrombocytopenia was the most predominant. The determinant factors that showed significant association with thrombocytopenia were Malaria Parasitic infection, one-year Inter-birth interval, History of abortion, History of hypertension, HIV infection, and HBV infection. Therefore, pregnant women should be continuously screened for thrombocytopenia to avoid excessive bleeding. Increasing Inter-birth interval, preventing abortion as well as timely diagnosis and treatment of underlying causes such as malaria infection, hypertension, HBV, and HIV is important to reduce the burden of thrombocytopenia.

**Supplementary Information:**

The online version contains supplementary material available at 10.1186/s12884-024-06609-6.

## Introduction

Thrombocytopenia is a condition in which the number of platelets decreases which happens due to increased platelet consumption and or decreased production of bone marrow megakaryocytes [1]. It is a type of hematological disorders that commonly occur during pregnancy and affects 5–10% of pregnant women and or the first few months after delivery [2]. It is a common occurrence during pregnancy, but it can also be a biomarker for a concomitant systemic or gestational illness, as well as a reason for maternal intervention or treatment that could harm the fetus [3].

The Platelate count decrement which is less than 150 × 109/L is one of physiological changes in pregnancy as compared with non-pregnant women, especially in the third trimester [4–6]. Around 8-13.5% are affected by thrombocytopenia due to gestational thrombocytopenia, of which 75% are due to a benign process. The thrombocytopenia among pregnant women in Africa and Ethiopia respectively reached 10.23% and 14.5 [5–7]. It can be either from increased body fluid due to hemodilution or due to increased platelet activation and accelerated destruction [8, 9].

The pathophysiology of thrombocytopenia is not clearly understood, but the studies suggest multiple factors to be the root cause of thrombocytopenia during pregnancy. It occurs either due to obstetric conditions, like gestational thrombocytopenia, and preeclampsia/eclampsia (2) or, secondary to systemic disorders like thrombocytopenic thrombotic purpura, and immune thrombocytopenia (3). The main causes associated with thrombocytopenia are rural lifestyles, human immunodeficiency virus (HIV) infection [10, 11], hepatitis B virus (HBV) infection [12], abortion, hypertension [13], lack of awareness (illiteracy), and increased gestational age [6]. About 8% of pregnant women with thrombocytopenia have a higher risk of hemorrhage or high loss of blood during or after childbirth, particularly when a ceserean section or other surgical intervention is needed during delivery [10, 14, 15].

Different studies reveal that thrombocytopenia can be caused by due to different causes [16]. The immune thrombocytopenic purpura is the first type of thrombocytopenia which is caused by an immune-mediated acquired disease of adults and children characterized by a transient or persistent decrease of the platelet count and, depending upon the degree of thrombocytopenia, increased risk of bleeding [17]. It occurs in one up to two of every 1,000 pregnancies and accounts for 5% of cases of pregnancy-associated thrombocytopenia [18].

The lower threshold of platelets, to define thrombocytopenia is 172.50-415.25 × 109/L whereas the lowest threshold of 100 × 109/L is used to define immune thrombocytopenia, which is observed in less than 1% of all pregnancies [19, 20]. The exact mechanism of the immune dysfunction is generally not known. Certain patients who have idiopathic thrombocytopenic purpura may have an indolent form of myelodysplasia that is not yet evident on bone marrow examination. Some difficulties in defining the pathology of immune thrombocytopenic purpura arise because of a heterogeneous disease with individual patients having different causes of thrombocytopenia and other difficulties related to the limited nature of assays, such as the antiplatelet antibody [21].

The second most common cause of thrombocytopenia is related to disorders of pregnancy such as pre-eclampsia or abruption-placenta [22, 23]. Approximately 7–15% of pregnant women caused by a variety of obstetric conditions such as incidental or gestational thrombocytopenia, disorders with increased platelet consumption pregnancy- induced hypertension syndrome, thrombotic thrombocytopenic purpura, drug-induced, systemic lupus erythematous, ant-phospholipid syndrome, disseminated intravascular coagulation. The diagnosis of specific disorders is often difficult because the time of onset of these disorders during pregnancy and their clinical manifestations often overlap [24–27].

Thrombocytopenia in pregnancy increases susceptibility to malaria and is associated with both plasmodium falciparum and plasmodium vivax increased chances of repeated infections. If these infections are treated appropriately, there are fewer chances of complications, but complicated malaria is associated with poor fetomaternal outcomes [28]. On the other hand, in patients, P. falciparum malaria lower platelet number in patients than in the controls [29].

Patients with eclampsia have more pregnancies complicated with thrombocytopenia than patients with both mild and severe forms of preeclampsia will manifest criteria for hemolysis, elevated liver enzymes, and low platelet counts syndrome [30]. A great number of disease processes or pregnancy-induced disorders can mask the correct diagnosis. Most patients have thrombocytopenia manageable through history, physical examination, laboratory evaluation, and appropriate consultation with obstetricians and hematologists [31].

Pregnant women are mostly affected by thrombocytopenia. Even though thrombocytopenia is a well-known hematological abnormality following anemia, only minimal studies have been done to determine the magnitude and associated factors. So aim of this study is to determine gestational thrombocytopenia and associated factors among pregnant women at Mizan Tepi University Teaching Hospital.

## Methodology

### Study area

The Antenatal Care Clinic (ANC) at Mizan Tepi University Teaching Hospital (MTUTH) was the site of the study. The hospital is situated in Mizan-Aman in the Southern Nations, Nationalities, and People’s Region (SNNPR), in southwest Ethiopia, 582 km from Addis Ababa, the country’s capital. A catchment population of 2.75 million persons is served by the hospital. For the people residing in Mizan-Aman town and its environs, the hospital offers a variety of inpatient and outpatient treatments [32]. It is the largest and the only teaching hospital region, with around six departments including Obstetrics and Gynaecology, pediatrics, surgery, internal medicine, pharmacy, and diagnostic departments (laboratories and radiology). ANC is one of the departments with its coordinator and professionals under Obstetrics and Gynecology. The clinic has full-time nurses, general practitioners, gynecologists, midwives, and Internists. The laboratory services given by the ANC clinic are urinalysis, stool microscopy, whole blood count, hematocrit, and hemoglobin determination.

### Study design and period

The cross-sectional study design was employed to assess the magnitude and associated factors of thrombocytopenia among pregnant women attending Antenatal care visits at MTUTH from September 2023 to November 2023.

### Source of population

All pregnant mothers who were attending ANC at MTU TH.

### Study population

All pregnant mothers who were attending ANC at MTUTH during the study period.

### Inclusion criteria

All Women who visited the antenatal care unit at MTUTH were able to provide blood samples for thrombocyte count and whose clinical data is recorded with full information.

### Exclusion criteria

Those individuals who were seriously ill or unable to provide blood samples, individuals with active bleeding, and seriously ill individuals were excluded during data collection.

### Sample size

The single population percentage formula was used to determine the necessary sample size for this investigation, taking into account the assumptions. According to a study, 18% of people had thrombocytopenia [33]. The following statistical procedure was used to establish sample size (n), which may be derived using an 18% proportion with a 95% confidence range and 5% marginal error.

1 $$n = \frac{{{{(Z\alpha /2)}^2}P\left( {1 - P} \right)}}{{{D^2}}}\,\,\,\,\,\,\,\,n = \frac{{{{\left( {1.96} \right)}^2}0.18\left( {1 - 0.18} \right)}}{{{{(0.05)}^2}}} = 230$$

The total sample size became 230 pregnant mothers who were on ANC follow-up during the study duration.

### Sampling techniques

To select study participants a systematic random sampling technique was used. 17 pregnant women on average attended ANC follow-up sessions each day. During the two-month data collection period, 732 pregnant were expected to visit the hospital ANC follow-up by considering only the working day of a month. The sampling interval (K) value was calculated by dividing the total number of pregnant during our study period by the sample size (732 / 230 = 3). Then to select the first participant of three we used the lottery method. Then study subjects were selected by every three individuals who are attending the ANC clinic of MTUTH.

### Variables

#### Dependent variable

Presence of thrombocytopenia (Yes/ No).

Independent variables (Socio-demographic and obstetric factors).

- Age.
- Gestational age.
- History of infection.
- Residence.
- HIV infection.
- Inter-birth interval.
- HBV infection.
- Malaria parasite infection.
- Education.
- History of DM.
- History of autoimmune disease.
- Meat consumption.
- Use green leafy vegetables.
- Gestational age.
- History of abortion.
- Abortion frequency.
- History of blood loss.
- History of hypertension.
- Alcohol consumption.

### Operational definitions

Thrombocytopenia: deficiency or decrement of platelets in the blood below 150.000/µL [8].

Moderate thrombocytopenia: deficiency of platelets in the blood between 50,000 to 99,000/µL [9].

Low/ mild thrombocytopenia: platelets in blood between 100,000 to 150,000/µL [9].

Normal platelet: platelets in blood between 150,000 to 450,000/µL [9].

Severe thrombocytopenia: deficiency platelets in blood is less than 50,000/µL [34].

### Data collection tool and approach

#### Sociodemographic and clinical data collection

The questionnaire was adapted from related studies on hematological disorders and parameters. Subsequently, it was translated into the Amharic local language, translated back into English, and verified for validity and consistency by three hematologists and language experts. The data was collected by structured questionnaire and collection was performed by trained expert nurses after permission or consent was obtained from pregnant women. The questionnaire had three parts including sociodemographic, and clinical for thrombocytopenia. The questionnaire was translated into the Amharic language. Sociodemographic data such as age, sex, residence, marital status, education, and religion were collected by using structured questionnaires via face-to-face interviews with study subjects.

### Sample collection procedures and hematological analysis

#### Blood collection procedures

About 3 ml of blood was collected with a sterile syringe and needle by an expert medical laboratory technologist into the study participant code number labeled EDTA anticoagulant test tube. The collected blood sample was delivered to the hematology laboratory for analysis of platelet parameters. The blood was transported to the hematology laboratory within 1 to 2 h and the analysis was performed. From the collected blood sample, hematological analysis was performed, and then a blood film was prepared from the remnant sample.

### Hematological analysis

The hematological analysis was performed on a blood sample in an EDTA anti-coagulated test tube to confirm the presence of thrombocytopenia by following standard operating procedures (Annex I). Platelet count was performed by were performed by using an automated hematology analyzer (Sysmex XP-300™ Automated Hematology Analyzer, United States of America (USA)). It was determined by flow cytometry or light scattering principle the blood sample is suspended in diluent and passes through the apparatus causing direct current resistance.

### Data quality assurance

Data collection tools were pretested on 5% of the sample groups of pregnant women who are following their Antenatal care service in Mizan Tepi University Teaching Hospital for consistency. The hematology analyzer was tested by using low, high, and normal controls.

### Data processing and analysis procedure

Data entry was performed into Epidata version 4.6 (Epidata, Inc. Redwood City, CA, United States) and the analysis was performed by using STATA (Software for statistics and data science) statistical software version 14 developed by Stata Corp for data. The principal investigator checked every day the collected data for completeness and accuracy. During the entry of data, it was cross-checked to ensure the right data was entered and cleaned for accuracy. Descriptive statistics including frequency, charts, tables, and percentages were used to summarize the data. The logistic regression model was fitted to determine the associations of independent variables with outcome variables. For measure of association for variables was analyzed by the bivariable logistic regression model and those variables that had a *P* value of 0.25 were included in a multivariable logistic regression model to control the confounding factors. Then multivariable logistic regression was computed for selected variables and the significance of the association was determined and interpreted. Both Crude odds ratio (COR) and adjusted odds ratio (AOR) with their corresponding 95% confidence interval (CI) were used to see the strength of the association between dependent and independent. A *p*-value < 0.05 in the multivariable logistic regression model was considered statistically significant. The words and tables were used to present the results. Based on the study result, conclusions and recommendations were made.

## Results

### Sociodemographic characteristics of study participants

In this study, a total of 230 pregnant mothers who were antenatal care attendants at MTUTH were included. One hundred thirty-two (57.39%) of the study subjects were aged < 27 years. Most of the study subjects (174 [76.65%]) lived in a rural residence (Table 1).

**Table 1:** Sociodemographic characteristics of pregnant women who attended antenatal care service at MTU TH in 2022 in southwest Ethiopia

Variables	Category	Frequency (*N*)	Percent (%)	
Age	18–27	132	57.39	
	28–36	82	35.65	
	37–45	16	6.96	
Residency	Urban	174	75.65	
	Rural	56	24.35	
Religion	Orthodox	93	40.43	
	Muslim	36	15.65	
	Protestant	96	41.74	
	Others ^a^	5	2.51	
Marital status	Single	20	8.7	
	Married	161	70	
	Divorced	41	17.83	
	Widowed	8	3.48	
Occupation	Farmer	17	7.4	
	Housewife	138	60	
	Merchant	19	8.3	
	Government employee	34	14.78	
	Others ^b^	22	9.56	
Educational Status	No formal education	63	27.4	
	Primary School	70	30.44	
	Secondary school	65	28.26	
	University/college	32	13.90	

Other in religion ^a^: Jehovah’s Witness, cultural religion, pagan

Other occupation ^b^: laborer, Non-governmental organization worker, driver

#### Prevalence of thrombocytopenia

**Fig. 1:**
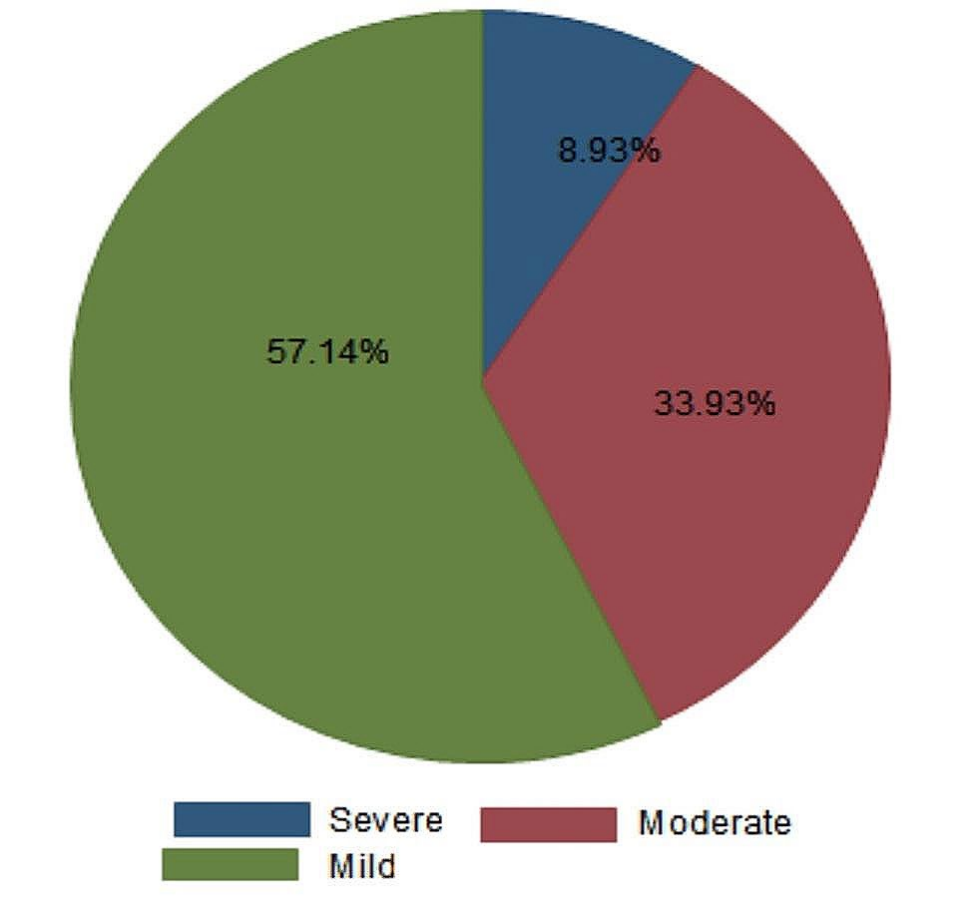
Pie chart indicating the severity of thrombocytopenia among pregnant women during the study period

The prevalence of thrombocytopenia among the study population was 24.35% (95% CI 23.19, 26.30) (Fig. 1).

### Correlated factor with thrombocytopenia

The mean platelet count with mean ± standard deviation platelet count was 223 × 109/L (± 104.5). The RBC, WBC, and Hgb have correlated factors with platelets. The correlation between them is positive which is a weakly positive correlation (Table 2).

**Table 2:** Correlated factors with thrombocytopenia of pregnant women who attended antenatal care service from September 2023 to November 2023 at MTU TH southwest Ethiopia

	Red blood cell	Platelets	Hemoglobin	leukocytes
Red blood cell	1.0000	0.0528	0.4182	0.4182
Platelets	0.0528	1.0000	0.1207	0.0774
Hemoglobin	0.4182	0.1207	1.0000	0.0247
Leukocyte	0.0148	0.0774	0.0247	1.0000

#### Factors associated with thrombocytopenia

To determine the association between the thrombocytopenia and independent variable bi-variable and multi-variable logistic regression model was used. Based on the analysis variables with *p* value less than 0.25 in the bivariable logistic regression model were included in the multivariable analysis. Accordingly, Malaria Parasitic infection, history of abortion, Meat consumption, history of hypertension, Birth interval, and family history of anemia showed significant association with thrombocytopenia (Table 3).

**Table 3:** Investigation of factors associated with thrombocytopenia among pregnant women by using bivariable and multivariable logistic regression model from September 2023 to November 2023 (*N* = 230)

Variables	Category		Thrombocytopenia		COR at CI (95%)	AOR at CI (95%)	*p*-value	
			Yes	No				
Residence	Urban		14 (25)	42 (75)	1	1		
	Rural		41 (21.56)	129 (74.14)	1.05 (0.87,1.8)	2.58 (0.59,11.20)	0.84	
Malaria Parasitic infection	Yes		116 (72.9)	43 (27.04)	9.27 (7.42, 10.87)		0.03	
	No		16 (22.5)	55 (77.5)	1			
History of DM	Yes		14 (11.96)	103 (88.4)	1.10 (0.44, 5.0)			
	No		8(10.96)	65 (89.04)	1		0.1	
	Unknown		5(12.5)	35(87.5)	1.16 (0.19, 7.69)			
History of autoimmune disease	Yes		7(28.0)	18(72)	1.27 (0.13,1.74)		0.17	
	No		48(23.4)	157(76.6)	1			
Meat consumption	At least /weak		8 ( 9.87)	73 (90.12)	1			
	Less often/ weak		22(25.29)	75 ( 74.71)	0.62 (0.13,2.86)	0.98(0.19, 5.0)		
	Never		26 (27.41)	46(75.71)	5.15(1.1, 13.2)	8.18(0.60,1.74)	0.25	
Use green leafy vegetables	At least per day		22 (20.2)	87 (79.80)	1			
	Less often per day		19 (15.7)	102 (84.3)	1.36 (0.25 4.4)		0.09	
Inter-birth interval	One		16 (33.34)	33 (68.75)	1.7 (1.24, 2.14)		0.004	
	Two		23 (25.27)	68 (74.72)	1.19 ( 0.98, 1.72)		0.3	
	Three		15 (23.40)	49 (76.6)	1.07 (0.93,1.43)		0.27	
	Four and above		6 (22.2)	21 (77.8)	1			
Gestational age	1st trimester		16 (22.2)	56 (77.8)	1			
	2nd trimester		18 (21.7)	65 (78.3)	1.03(0.74, 2.58)	1.48 (1.1, 1.93)	0.02	
	3rd trimester		16 (21.34)	59 (78.64)	1.05 (0.51, 20.18)	1.73 (0.8, 3.31)	0.16	
History of abortion	Yes		24 (36.96)	41 (93.08)	2.43 (5.25, 67.4)	3.94(1.13, 4.26)	0.04	
	No		32 (19.4)	133 (80.6)	1	1		
Abortion frequency	Once at a time		7 (19.44)	29(80.6)	1		0.23	
	Twice at a time		9 (31.03)	20 (68.97)	0.55 (0.23,1.34)			
history of blood loss	Yes		24 (23.76)	77 (76.24)	1.01(0.70, 2.17)	1.44 (0.37, 5.61)	0.61	
	No		31 (24.03)	98 (75.97)	1			
History of hypertension	Yes		27(30.68)	61(69.32)	1.5 (1.29, 2.5)	3.12 (1.56, 4.12)	0.01	
	No		27 (23.30)	89 (76.7)	1			
	I do not know		6(23.1)	20 (76.9)	1.51(0.33, 6.92)	1.80(0.375, 8.55)	0.45	
Alcohol consumption	Yes		21 (23.6)	68 (76.40)	1.37 (0.67,1.87)	1.87(0.85,1.5)		0.12
	No		26 (18.44)	115 (81.56)	1			
HIV	Yes		6 (35.30)	11 (64.70)	1.81 (1.32.2.52)	2.14 (1.87, 2.62)		0.02
	No		49(23.11)	163 (76.89)	1	1		
HBV	Yes		8 (42.10)	11(57.80)	2.65 (2.2, 2.8)	3.0 ( 2.82,3.34)		0.04
	No		47 (22.30)	171(77.70)	1			

## Discussion

Thrombocytopenia is characterized by an abnormally low thrombocyte count in the bloodstream, which can lead to blood disorders and spontaneous bleeding. It can happen when there is disseminated intravascular coagulation, and leukemia has been linked to decreased platelet synthesis and platelet destruction. Some infections and autoimmune diseases including systemic lupus erythematosus (SLE) and idiopathic thrombocytopenic purpura (ITP), which causes thrombocytopenia, may be among them [3].

In this study, the prevalence of thrombocytopenia among pregnant women was 24.35% [95% CI 23.19–26.30]. The finding of this study was greater than studies conducted in Gondar [Ethiopia] [8.8%] [35], Dessie [Ethiopia] [9.9%] [6], Wolkite [Ethiopia] [14.5%] [7], Harar [Ethiopia] [14.1%] [36], Debre birhan [Ethiopia] [10.2%] [37], Ghana [15.3%], Cameroon [8.9%], Nigeria [13.5%], Tripoli [18%], India [8.8%], Iraq [8.6%], Pakistan [8%], Lahore [Pakistan] [16.86%] [9, 27, 37–44] and lower than the studies conducted in China (28.2%) [45]. The difference might be due to socio-demographic differences in the study subject, differences in the study design, geographical variation, availability and accessibility of health care facilities, health-seeking behaviours, genetic variation related to pregnancy, a diagnostic method used, and differences in dietary habits of the study populations.

Among the thrombocytopenic pregnant women in this study, 32(57.14%) had mild, 19 (33.93%) had moderate, and 5 (8.93%) had severe thrombocytopenia. The finding of this study is in line with the study done at Dessie (4% severe) but in another way, the study at Dessie indicated that there were no findings on moderate type of thrombocytopenia, and higher mild thrombocytopenia (76%) [7]. The finding of this study was inconsistent with the study done in Ghana which showed 8.8% of moderate thrombocytopenia [27]. The finding of this study also indicated the severe form of thrombocytopenia was higher than that of a study done in Tripoli [Libya] which showed that there was no severe thrombocytopenia, but there were 78.5% of mild, and 21.5% of moderate thrombocytopenia [41]. The difference may be sample size and study area variation with this study.

According to the findings of this study, people with malaria parasitic infection were 9.27 times (AOR 9.27 at 95% CI 7.42, 10.87) at risk of developing thrombocytopenia than individuals who were not infected. The possible reason might be malaria may result in both immunological and non-immunological disturbances including coagulation, splenic sequestration, and immune-mediated platelet destruction that results in thrombocytopenia [46, 47].

This study finding revealed that pregnant women with women who had a birth history with a one-year delivery interval were 1.7 times (AOR 1.7 at 95% CI 1.24, 2.14), at risk of developing thrombocytopenia than individuals who give birth at four years and above birth interval. This might be due to the presence of postpartum hemorrhage especially in the case of cesarean delivery which results in increased activation and depletion of platelets which finally causes thrombocytopenia [48].

As the finding of this study, pregnant women with a history of abortion were 3.94 times (AOR 3.94, 95% CI 3.13, and 4.86) at risk of developing thrombocytopenia than those without a history of abortion. The reason for this might be abortion results in serious bleeding which causes increased platelet consumption secondary to thrombosis [49].

Additionally, this study finding indicated that pregnant women history of hypertension 3.12 times (AOR 3.12 95% CI 1.56, 4.12) than those with no history of hypertension. The possible reason for this risk of thrombocytopenia is the pathology of hypertensive disorders of pregnancy, such as preeclampsia, eclampsia, and pre-eclampsia superimposed on chronic hypertension and related complications, such as haemolyses, elevated liver enzymes, low platelets syndrome, and acute fatty liver disease, maybe the cause of this risk of thrombocytopenia [13, 47].

This study finding revealed that pregnant women with women who have HIV infection 1.81 times (AOR 1.81 95% CI 1.32.2.52) are at risk of developing thrombocytopenia than individuals who have not been infected by HIV. The reason that HIV-infected pregnant women are at risk of thrombocytopenia might be that HIV infection affects and depletes hematopoietic stem cells then causes thrombocytopenia. In addition to this, the mechanism might be immune-mediated destruction of platelets by antibodies, diminished production of megakaryocytes due to infection, hypersplenism, malignancy, and myelosuppression effects of HIV medications as well as viral negative factors (proteins) [11, 50].

The finding of this study indicated that pregnant women with HBV infection were 3 times (AOR 3.0 95% CI 2.82, 3.34) at risk than individuals who have not been infected by HBV. The possible reason for the occurrence of thrombocytopenia due to HBV infection might be due to its results in hepatic impairment and cirrhosis which inhibits decreased protein production and platelet [10, 12].

### Limitations of the study

Since this study is a cross-sectional study design, may not get full information on cause and effect or the temporal relationship between cause and effect or, it did not allow us to observe causality in the relationship between thrombocytopenia and its associated factors, as it is a temporal association. We got many variables that may indicate association with the prevalence of thrombocytopenia which were suggested as the cause for thrombocytopenia but were not written and supported by another article or scientific studies and resulted in limited comparison across the world.

### Conclusion and recommendation

The magnitude of thrombocytopenia was 24.35%, and mild type of thrombocytopenia was the most predominant. The determinant factors that showed significant association with thrombocytopenia were Malaria Parasitic infection, one-year birth interval, History of abortion, History of hypertension, HIV infection, and HBV infection. Platelet count assessment should be done regularly and performed as a routine laboratory test during antenatal care to monitor the status of pregnant mother by health care providers to screen for thrombocytopenia to avoid excessive bleeding during pregnancy and or delivery, especially in women who have a history of malaria parasite infection, one-year inter-birth interval (interpregnancy intervals), and history of abortion, hypertension, HIV infection, and HBV infection. Preventing abortion, hypertension, malaria infection, HBV, HIV, and increasing inter-birth interval as well as timely diagnosis and treatment of underlying causes is important to reduce the burden of thrombocytopenia among pregnant women. Government officials and non - non-governmental organization workers working on pregnant women should pay attention to prevent thrombocytopenia and to achieve favorable feto-maternal outcomes during gestation and delivery. Appropriate follow-up should implemented during new-born delivery for women with severe thrombocytopenia to prevent bleeding complications.

### Electronic supplementary material

Below is the link to the electronic supplementary material.

Supplementary Material 1

## Data Availability

All relevant data are available within the manuscript. In case of need, the data that support the findings of this study are available from the corresponding author on reasonable request. Data is provided within the manuscript or supplementary information files as annexes.

## Abbreviations

ANC: Antenatal care
HIV: Human immune deficiency virus
HBV: Hepatitis B Virus
DM: Diabetes, Mellitus
WBC: White blood cell
RBC: Red blood cell
Hgb: Hemoglobin
MTU TH: Mizan Tepi University Teaching Hospital

## References

[1] Erkurt MA, Kaya E, Berber I, Koroglu M, Kuku I (2012). Thrombocytopenia in adults. J Hematol.

[2] Cines DB, Levine LD. Thrombocytopenia in pregnancy. *Hematology 2014, the American Society of Hematology Education Program Book* 2017, 2017(1):144–151. 10.1182/asheducation-2017.1.144 PMC6142617 29222249

[3] Silva CL, Grando AC. Complications of idiopathic thrombocytopenic purpura in pregnancy: a review of literature. Jornal Brasileiro De Patologia E Med Laboratorial. 2021;57.

[4] Santoshi RK, Patel R, Patel NS, Bansro V, Chhabra G, Chhabra GS. A comprehensive review of thrombocytopenia with a spotlight on intensive care patients. Cureus. 2022;14(8). 10.7759/cureus.27718 PMC9356658 35949449

[5] Getawa S, Getaneh Z, Melku M. Thrombocytopenia among pregnant women in Africa: a systematic review and meta-analysis. Pan Afr Med J. 2022;41(1). 10.11604/pamj.2022.41.334.30175 PMC9268328 35865846

[6] Ebrahim H, Kebede B, Tilahun M, Debash H, Bisetegn H, Tesfaye M (2022). Magnitude and associated factors of thrombocytopenia among pregnant women attending antenatal care clinics at Dessie comprehensive specialized hospital, Northeast Ethiopia. Clin Appl Thromb Hemost.

[7] Haile K, Kebede S, Abera T, Timerga A, Mose A. Thrombocytopenia among pregnant women in southwest Ethiopia: burden, severity, and predictors. J Blood Med. 2022;275–82. 10.2147/JBM.S365812 PMC9150712 35651987

[8] Chandra S, Tripathi AK, Mishra S, Amzarul M, Vaish AK (2012). Physiological changes in hematological parameters during pregnancy. Indian J Hematol Blood Transfus.

[9] Asrie F, Enawgaw B, Getaneh Z (2017). Prevalence of thrombocytopenia among pregnant women attending antenatal care service at Gondar University Teaching Hospital in 2014, northwest Ethiopia. J Blood Med.

[10] Kebede SS, Yalew A, Yesuf T, Melku M, Bambo GM, Woldu B (2022). The magnitude and associated factors of immune hemolytic anemia among human immuno deficiency virus infected adults attending University of Gondar comprehensive specialized hospital north west Ethiopia 2021 GC, cross sectional study design. PLoS ONE.

[11] Lv X, Li P, Yue P, Tang P, Zhou F (2023). Risk factors and prognosis of thrombocytopenia in people living with HIV/AIDS. Therapeutic Adv Hematol.

[12] Yang YT, Wang LL, Yan LT, Zhang LT, Zhou W, Chen QF, Chen Y, Zheng SJ, Duan ZP, Li JF (2020). Platelet count is closely associated with the severity of liver injury in patients with chronic hepatitis B virus infection: a cross–sectional study. Experimental Therapeutic Med.

[13] Deshmukh V, Nasrin A, Gadappa S. Thrombocytopenia in hypertensive disorder of pregnancy: maternal and perinatal outcome. Parity, 77:23.

[14] Gernsheimer T, James AH, Stasi R (2013). How I treat Thrombocytopenia in pregnancy. Blood J Am Soc Hematol.

[15] Mangla A, Hamad H. Thrombocytopenia in Pregnancy. *StatPearls* 2021. 31613485

[16] Smock K, Perkins S (2014). Thrombocytopenia: an update. Int J Lab Hematol.

[17] Kelton JG (2002). Idiopathic thrombocytopenic purpura complicating pregnancy. Blood Rev.

[18] Salnlo S, Kekomäki R, Rllkonen S, Teramo K (2000). Maternal thrombocytopenia at term: a population-based study. Acta Obstet Gynecol Scand.

[19] Bimerew LG, Demie T, Eskinder K, Getachew A, Bekele S, Cheneke W, Sahlemariam Z, Adisu W, Asres Y, Yemane T (2018). Reference intervals for hematology test parameters from apparently healthy individuals in southwest Ethiopia. SAGE open Med.

[20] Cines DB, Levine LD (2017). Thrombocytopenia in pregnancy. Blood.

[21] Cooper N, Bussel J (2006). The pathogenesis of immune thrombocytopaenic purpura. Br J Haematol.

[22] Greer IA (2003). Thrombophilia: implications for pregnancy outcome. Thromb Res.

[23] Kam P, Thompson S, Liew A (2004). Thrombocytopenia in the parturient. Anaesthesia.

[24] Parnas M, Sheiner E, Shoham-Vardi I, Burstein E, Yermiahu T, Levi I, Holcberg G, Yerushalmi R (2006). Moderate to severe thrombocytopenia during pregnancy. Eur J Obstet Gynecol Reproductive Biology.

[25] Belayneh FG, Mariam A, Solomon F (2015). The prevalence of thrombocytopenia, and associated factors among pregnant women attending antenatal care at Hawassa University Referral Teaching and Hospital. J Harmoniz Res Med Hlth Sci.

[26] Saeed HD, Sabri EH, Muhammad EM (2018). The prevalence of thrombocytopenia in pregnant women in Baghdad: single centre study. J Fac Med.

[27] Olayemi E, Akuffo FW. Gestational thrombocytopenia among pregnant Ghanaian women. Pan Afr Med J. 2012;12(1). PMC3415055 22891092

[28] Briggs R, Chari RS, Mercer B, Sibai B (1996). Postoperative incision complications after cesarean section in patients with antepartum syndrome of hemolysis, elevated liver enzymes, and low platelets (HELLP): does delayed primary closure make a difference?. Am J Obstet Gynecol.

[29] Adam MB, Adam GK, Rayis DA, Elbashir MI, Adam I (2012). Thrombocytopenia in pregnant women with Plasmodium Falciparum malaria in an area of unstable malaria transmission in eastern Sudan. BMC Clin Pathol.

[30] Sullivan CA, Martin JN (1995). Management of the obstetric patient with thrombocytopenia. Clin Obstet Gynecol.

[31] Levy JA, Murphy LD (2002). Thrombocytopenia in pregnancy. J Am Board Family Pract.

[32] Marye M, Yosef T (2022). Determinants of health professionals’ engagement at Mizan-Tepi University Teaching Hospital in Southwest Ethiopia. Heliyon.

[33] Elgodwi S, Lawar M, Saadallah A (2021). Prevalence of thrombocytopenia among pregnant women in Tripoli region/Libya. Int J Biomed Sci Eng.

[34] Myers B (2012). Diagnosis and management of maternal thrombocytopenia in pregnancy. Br J Haematol.

[35] Asrie F, Enawgaw B, Getaneh Z. Prevalence of thrombocytopenia among pregnant women attending antenatal care service at Gondar University Teaching Hospital in 2014, northwest Ethiopia. J Blood Med. 2017;61–6. 10.2147/JBM.S136152 PMC5479266 28670146

[36] Arkew M, Asmerom H, Sileshi B, Walle M, Adane A, Ayele F, Tebeje F, Bete T, Ersino T, Gemechu K (2024). Thrombocytopenia and associated factors among pregnant women attending antenatal care at Hiwot Fana Comprehensive Specialized Hospital, Eastern Ethiopia: a cross-sectional study. Medicine.

[37] Shitie D, Zewde T, Molla Y (2018). Anemia and other hematological profiles of pregnant women attending antenatal care in Debre Berhan Referral Hospital, North Shoa, Ethiopia. BMC Res Notes.

[38] Muhammad EM, Sabri EH, Saeed HD (2018). The prevalence of thrombocytopenia in pregnant women in Baghdad: single centre study. J Fac Med Baghdad.

[39] Nisha S, Amita D, Uma S, Tripathi A, Pushplata S (2012). Prevalence and characterization of thrombocytopenia in pregnancy in Indian women. Indian J Hematol Blood Transfus.

[40] Shamoon RP, Muhammed NS, Jaff MS (2009). Prevalence and etiological classification of thrombocytopenia among a group of pregnant women in Erbil City, Iraq. Turk J Hematol.

[41] Elgodwi S, Lawar M, Saadallah A (2021). Prevalence of Thrombocytopenia among pregnant women in Tripoli Region/Libya. Int J Biomedical Sci Eng.

[42] Mbanya D, Tagny CT, Takoeta E, Mbu R, Kaptue L (2008). Factors associated with thrombocytopenia among pregnant women in Cameroon. Cahiers d’études et de recherches francophones/Santé.

[43] Brohi ZP, Perveen U, Sadaf A. Thrombocytopenia in pregnancy: an observational study. Pakistan J Med Res. 2013;52(3).

[44] Ijaz T, Atif M, Ullah M, Arshad S, Ashraf S, Munir S, Imran M, Ahmed F, Imran M, Fatima Z (2016). Prevalence of Anemia and Thrombocytopenia in pregnant females of Lahore. Life sci-Int J.

[45] Wang X, Xu Y, Luo W, Feng H, Luo Y, Wang Y, Liao H (2017). Thrombocytopenia in pregnancy with different diagnoses: differential clinical features, treatments, and outcomes. Medicine.

[46] Rasheed A, Saeed S, Khan S (2008). Platelet count in malaria. Pakistan J Phytopathol.

[47] Katke RD, Gohil DP (2014). Thrombocytopenia during pregnancy: an institutional based study. Int J Reprod Contracept Obstet Gynecol.

[48] van Dijk WE, Nijdam JS, Haitjema S, de Groot MC, Huisman A, Punt MC, Evers AC, Schutgens RE, Lely AT, van Galen KP (2021). Platelet count and indices as postpartum hemorrhage risk factors: a retrospective cohort study. J Thromb Haemost.

[49] Kosus N, Kosus A, Yildirim M, Duran M, Turhan N. Mean platelet volume as a marker of thrombosis in patients with missed abortion. Acta Haematol. 2011;125(4). 10.1159/000322943 21252498

[50] Getawa S, Aynalem M, Bayleyegn B, Adane T (2021). The global prevalence of thrombocytopenia among HIV-infected adults: a systematic review and meta-analysis. Int J Infect Dis.

