# Supplementary material for: Magnitude and associated factors of thrombocytopenia, among pregnant women at Mizan Tepi university teaching hospital south west Ethiopia
Source: BMC Pregnancy Childbirth. 2024 Jun 7;24:411. doi: 10.1186/s12884-024-06609-6 (PMC11157699; doi:10.1186/s12884-024-06609-6)
Supplement: Supplementary file 1 — Supplementary Material 1 [file 12884_2024_6609_MOESM1_ESM.docx]

# **Annexes**

## **Annex I: English Version Information sheet and Consent form**

**Part I:** **Information sheet**

**Introduction**: My name is Samuel Sahile I am lecturer of Mizan Tepi university department of Medical Laboratory Sciences I am going to conduct a survey on magnitude of thrombocytopenia and associated factors in thrombocytopenia patients.

**Study title**: The Magnitude and associated factors of thrombocytopenia, among pregnant women at Mizan Tepi university teaching hospital south west Ethiopia..

**Objective of the study:** the aim of this study is to determine the Magnitude and associated factors of thrombocytopenia, among pregnant women at Mizan Tepi university teaching hospital south west Ethiopia.

**Benefit of this study:** Conducting this study will be used to advance the diagnosis of thrombocytopenia in these individuals and know the current status of the problems. If you have anemia especially thrombocytopenia you will be linked to ANC clinicians with your laboratory results for additional diagnosis and treatment of disease. The study output (result) will be used for planning health programs and policy makers for reduction of anemia as well as thrombocytopenia. You understand that you will not get any financial benefit. Your cooperation and willingness to the study will be very helpful in understanding current prevalence of problem.

**Risks of this study for participants**: There may be little pain during blood sample collection but do not cause any health harm or long live abnormality.

**Rights and privacy**: Your name will not be written in the form and I assure you that all the information will be kept strictly confidential. Your participation is voluntary based and you are not obligated to participate. You should know that the information and blood sample that going to be used for this study only. All the information given for the study and the results are confidential. If you are not comfortable please feel free to refuse. Therefore, with full understanding of the situations you agree to give the entire necessary information **a**nd blood sample for laboratory analysis.

**Person to contact;** please direct any questions or problems you may encounter during this study to the principal investigator. Address; [+251917768545/samissahile45@gmail.com](mailto:+251917768545/samissahile45@gmail.com)

**Informed consent**

I participant undersigned the purpose of the study titled as magnitude of thrombocytopenia in pregnant women at MTUTH from September 2023 to November 2023 G C. I have been informed there is no harm except little discomfort during sample collections. I have been informed that other people will not know my results. I understand that there is no benefit to me personally apart from clinical service I get from these results .I have been told that participation in this study is voluntary and I may refuse to be in the study. The study has been explained to me in the language I understand. I give consent to participate after a clear understanding of the objectives and conditions of the study.

Participant’s name ----------------------------Signature----------------------- Date: ----------------------Data collector name: --------------------------- Signature: ---------------------- Date: -----------------Name of investigator _____________Signature ___________Date of investigation __________

**Informed assent**

I guardian undersigned the purpose of the study titled as magnitude of thrombocytopenia in pregnant patient at MTUTH from September 2023 to November 2023 G C. I have been informed there is no harm except little discomfort during sample collections. I have been informed that other people will not know my patients results. I understand that there is no benefit to me personally apart from clinical service he/she get from these results .I have been told that participation in this study is voluntary and I may refuse as my patient want to refuse to be participant in the study. The study has been explained to me in the language I understand. I give consent to participate after a clear understanding of the objectives and conditions of the study.

Guardians’ name --------------------------------------------

Relationship to participant: -----------------Signature----------------------- Date: ------------------------

Data collector name: --------------------------- Signature: ---------------------- Date: -----------------

Name of investigator _____________Signature ___________Date of investigation __________

**ID.No:______________**

| Socio –demographic ,clinical and nutritional information of pregnant women | | | |
| --- | --- | --- | --- |
| S no | Socio –demographic questions please encircle in correct information you have. | | |
| 01 | How old are you? Age in years |  | |
| 02 | Where do you live?(Residence) | Urban | 1 |
|  |  | Rural | 2 |
| 03 | What is your occupation? | Farmer | 1 |
|  |  | House wife | 2 |
|  |  | Merchant | 3 |
|  |  | Governmental employee | 4 |
|  |  | Others | 5 |
| 04 | Religion | Orthodox | 1 |
|  |  | Muslim | 2 |
|  |  | Protestant | 3 |
|  |  | Others | 4 |
| 05 | How much is your monthly income? | |  |
| 06 | What is your educational Status? | No formal education | 1 |
|  |  | Primary School | 2 |
|  |  | Secondary school | 3 |
|  |  | University/college | 4 |
| 07 | Marital status | Single | 1 |
|  |  | Married | 2 |
|  |  | Divorced | 3 |
|  |  | Widowed | 4 |
|  | | | |

B) Clinical data of participant

| 08 | Frequency of have you given birth | One year | 1 |  |  |  |
| --- | --- | --- | --- | --- | --- | --- |
|  |  | Two year | 2 |  |  |  |
|  |  | Three | 3 |  |  |  |
|  |  | Four and above | 4 |  |  |  |
| 09 | History of hypertension | Yes | 1 |  |  |  |
|  |  | No | 2 |  |  |  |
| 10 | History of DM |  |  |  |  |  |
| 11 | Have you past history TB/ bacterial disease | Yes | 1 |  |  |  |
|  |  | No | 2 |  |  |  |
| 12 | Malaria parasite infection | Yes | 1 |  |  |  |
|  |  | No | 2 |  |  |  |
| 13 | HIV infection | Yes | 1 |  |  |  |
|  |  | No | 2 |  |  |  |
| 14 | HBV infection | Yes | 1 |  |  |  |
|  |  | No | 2 |  |  |  |
| 15 | Family history of hemolysis(anemia) | Yes | 1 |  |  |  |
|  |  | No | 2 |  |  |  |
|  |  | I don’t know | 3 |  |  |  |
| 16 | History of auto immune disease | Yes | 1 |  |  |  |
|  |  | No | 2 |  |  |  |
| 17 | Neoplastic diseases | Yes | 1 |  |  |  |
|  |  | No | 2 |  |  |  |
| 18 | History of abortion | Yes | 1 |  |  |  |
|  |  | No | 2 |  |  |  |
| 19 | If question number 18 yes frequency of abortion | Yes | 1 |  |  |  |
|  |  | No | 2 |  |  |  |
| 20 | Gestational age | 1^st^ trimester | 1 |  |  |  |
|  |  | 2^nd^ trimester | 2 |  |  |  |
|  |  | 3^rd^ trimester | 3 |  |  |  |
| Nutrional data’s of participant | | | |  |  |  |
| 21 | Did you drink tea or coffee after meal? | Yes | 1 |  |  |  |
|  |  | No | 2 |  |  |  |
| 22 | Do you eat meat | Yes | 1 |  |  |  |
|  |  | No | 2 |  |  |  |
| 23 | If answer for question 20 is yes how many times | Daily | 1 |  |  |  |
|  |  | Every two day | 2 |  |  |  |
|  |  | Every two week | 3 |  |  |  |
|  |  | Once a month | 4 |  |  |  |
| 24 | Do you eat vegetable | Yes | 1 |  |  |  |
|  |  | No | 2 |  |  |  |
| 25 | If answer for question 16 is yes how many times | Daily | 1 |  |  |  |
|  |  | Every two day | 2 |  |  |  |
|  |  | Every two week | 3 |  |  |  |
|  |  | Once a month | 4 |  |  |  |
| 26 | Have you taken iron /folate | Yes | 1 |  |  |  |
|  |  | No | 2 |  |  |  |
| 27 | Alcohol consumption | Yes | 1 |  |  |  |
|  |  | No | 2 |  |  |  |

Laboratory results ID.No**:______________**

| Hematologic profiles | Mean | SD | Maximum | Minimum |
| --- | --- | --- | --- | --- |
| Total leukocyte |  |  |  |  |
| Neutrophil |  |  |  |  |
| Eosinophil |  |  |  |  |
| Basophil |  |  |  |  |
| Monocyte |  |  |  |  |
| Lymphocyte |  |  |  |  |
| Erythrocyte |  |  |  |  |
| RDW ,f l |  |  |  |  |
| Platelet |  |  |  |  |

## **Annex III; Dummy table**

# **Table I: - Age and Sex distribution of magnitude of thrombocytopenia in pregnant patient at MTUTH from September 2023 to November 2023 G C.**

| Age | Sex | | Total |
| --- | --- | --- | --- |
|  | Male | Female |  |
| 15-30 |  |  |  |
| 31-45 |  |  |  |
| 41-64 |  |  |  |
| >64 |  |  |  |
| Total |  |  |  |

Table II; - Distribution of magnitude of thrombocytopenia in pregnant patient at MTUTH from September 2023 to November 2023 G C

| Sr. No | Marital status | Thrombocytopenia | | Total |
| --- | --- | --- | --- | --- |
|  |  | Yes | No |  |
| 1 | Married |  |  |  |
| 2 | Single |  |  |  |
| 3 | Divorced |  |  |  |
| 4 | Widowed |  |  |  |
| Total | |  |  |  |

Table III: distribution of magnitude of thrombocytopenia in pregnant patient at MTUTH from September 2023 to November 2023 G C

| Sr. No | Occupation | Thrombocytopenia | | Total |
| --- | --- | --- | --- | --- |
|  |  | Yes | No |  |
| 1 | Student |  |  |  |
| 2 | Employer |  |  |  |
| 3 | House wife |  |  |  |
| 4 | Laborer |  |  |  |
| 5 | Farmer |  |  |  |
| 6 | Others |  |  |  |

Table IV; - Distribution of **thrombocytopenia in pregnant patient at MTUTH from September 2023 to November 2023 G C**

| Sr. No | Locality | Thrombocytopenia | | Total |
| --- | --- | --- | --- | --- |
|  |  | Yes | No |  |
| 1 | Urban |  |  |  |
| 2 | Rural |  |  |  |
| 3 | Total |  |  |  |

Table VII; - Distribution of magnitude of thrombocytopenia in pregnant patient at MTUTH from September 2023 to November 2023 G C

| History | | | | **Yes** | **No** | **Total** |
| --- | --- | --- | --- | --- | --- | --- |
| Past opportunistic illnesses | | | |  |  |  |
| History of blood loss | | | |  |  |  |
| Other Medication (antibiotics) | | | |  |  |  |
| History transfusion | | | |  |  |  |
| Family history of hemolytic anemia | | | |  |  |  |
| Malaria parasite infection | | | |  |  |  |
| History of DM | | | |  |  |  |
| Gestational age | 1^st^ | | |  |  |  |
|  | 2^nd^ | | |  |  |  |
|  | 3^rd^ | | |  |  |  |
| History of abortion | | | |  |  |  |
| Frequency of abortion | | | |  |  |  |
| Birth interval in year | | One year | |  |  |  |
|  |  | Two- | |  |  |  |
|  |  | Three | |  |  |  |
|  |  | Four and above | |  |  |  |
| History of hypertension | | | Yes |  |  |  |
|  |  |  | No |  |  |  |
| Auto immune disease | | | Yes |  |  |  |
|  | | | No |  |  |  |
| Alcohol consumption | | | Yes |  |  |  |
|  |  |  | No |  |  |  |
| HIV | | | Yes |  |  |  |
|  |  |  | No |  |  |  |
| HBV | | | Yes |  |  |  |
|  | | | No |  |  |  |

Table VIII - Distribution of magnitude of thrombocytopenia in pregnant patient at MTUTH from September 2023 to November 2023 G C.

| Take iron or folic supplement | | | | Thrombocytopenia | | | Total |
| --- | --- | --- | --- | --- | --- | --- | --- |
|  |  |  |  | Yes | No | |  |
| Yes | | |  |  |  |  | |
| No | | |  |  |  |  | |
| Meat consumption | Yes | |  |  |  |  | |
|  | No | |  |  |  |  | |
| **Have tea or coffee immediately after meal** | | | |  |  |  | |
| Yes | | |  |  |  |  | |
| No | | |  |  |  |  | |
| Use green leafy vegetables | | Yes |  |  |  |  | |
|  |  | No |  |  |  |  | |

**Annex IV; Materials and reagents**

- Cotton
- Pencil
- Absolute Ethanol
- Sysmex hematology analyser
- Glove
- Test tube
- 3 or 4 ml Purple vacationer tube
- Needle
- Vacationer holder
- Alcohol swab (70%)
- Cotton balls
- Normal saline
- Sysmex reagent.
- Mixer
- New methylene /Brilliant Cresol Blue
- Wright satin
- Heparinized/EDTA capillary tube
- Disposable glove
- Tourniquet
- Microscope with 100X objective
- Microscope slides
- Glass test tubes
- Centrifuge
- Plastic pasture pipette
- Distilled water
- Water bath
- Oil immersion

## **Annex V; - Principles and Laboratory procedure**

**1. Sample procedure**

**Venous blood collection**

1. Assemble all necessary equipment
2. Identify the patient and label the test tubes by patient’s identification number.
3. Visually inspect and choose the arm from cephalic, basilic, and median cubital veins that was not repeatedly used for venipuncture, free of bruises, abrasions, and sites of infection.
4. Apply the tourniquet
5. Using a cotton ball saturated with 70% alcohol clean the skin in the area of the venipuncture.
6. Allow the site to dry
7. Use one hand to hold the evacuated tube or syringe and one or more fingers of the other hand to secure the skin area of the forearm below the intended venipuncture site.
8. Hold the needle with attached syringe or evacuated tube at angle of 20°about 1 to 2 inches below and in a straight line with the intended venipuncture site.
9. Gently insert the needle through the skin and into the vein.
10. Release tourniquet as soon as the blood begins to flow into the evacuated tube
11. After the desired amount of blood has been drawn place a gauze pad over the venipuncture site.
12. Withdraw the blood collecting unit with one hand and immediately press down on the gauze pad and elevate the entire arm.
13. Place a non-allergenic adhesive spot or strip over the venipuncture site
14. Mix tubes with anticoagulant by inverting the tubes several times.
15. Clean up supplies from the work area, remove gloves, and wash hands.

**2. Complete Blood Count by hematology analyzer (Sysmex )**

**Principle**

The Sysmex analyzer is a quantitative, automated hematology analyzer for in-vitro diagnostic use in screening patient populations in clinical laboratories. The blood sample, which is suspended in diluted sample, will pass through the apparatus causing DC resistance. As this occur change in blood cell size is detected as the electrical pulse and blood cell count is calculated by counting pulse. The Sysmex Analyzer provides the following: CBC, Leukocyte 5-Part Differential (Diff), Reticulocyte, and Nucleated RBC on whole blood. A cyanide-free CBC lytic reagent that lyses RBC for the white blood cell count, and works in conjunction with Coulter Sysmex diluent to generate a stable hemoglobin measurement and used to lyse the RBC and discriminates nucleated RBC from white blood cells.

The reticulocyte stain reagent is a cyanide-free reagent that uses a dye to stain reticulocytes. The reticulocyte-clearing reagent is a cyanide-free reagent that stabilizes the dye-reticulum complex to enhance discrimination of reticulocytes from mature RBC utilizing the volume, conductivity and scatter technology. Sysmex Cleaner is a cyanide-free, aldehyde-free cleaning agent that degrades residual materials so that they may be flushed from the system with the diluent.

**Specimen requirements**

About 3-4 ml of venous blood collected into EDTA tubes.

**Procedure**

- - - 1. Turn on the power switch on the front side of the analyzer.
      2. Perform quality control analysis on 3 levels of control blood material (low, normal and high) to verify that the instrument is performing within the specified ranges
      3. Specimen will be collected into EDTA (purple) vacutainer (2 or 3 ml volume).
      4. Well mix blood with EDTA and perform CBC and reticulocyte count.
      5. After the specimen processing module cycles the samples, review the sample results at the system manager.
      6. Finally print the output and register
